# REST-Governed Gene Expression Profiling in a Neuronal Cell Model Reveals Novel Direct and Indirect Processes of Repression and Up-Regulation

**DOI:** 10.3389/fncel.2015.00438

**Published:** 2015-11-10

**Authors:** Jose M. Garcia-Manteiga, Silvia Bonfiglio, Lucrezia Folladori, Maria L. Malosio, Dejan Lazarevic, Elia Stupka, Davide Cittaro, Jacopo Meldolesi

**Affiliations:** ^1^Center for Translational Genomics and Bioinformatics, Scientific Institute San RaffaeleMilan, Italy; ^2^CNR Institute of Neuroscience and Humanitas Clinical and Research CentreMilan, Italy; ^3^Division of Neurosciences, Vita-Salute San Raffaele University and Scientific Institute San RaffaeleMilan, Italy

**Keywords:** RNA-Seq, PC12 clones, different REST levels, cooperative transcription factors, differential gene expression, gene repression and up-regulation

## Abstract

The role of REST changes in neurons, including the rapid decrease of its level during differentiation and its fluctuations during many mature functions and diseases, is well established. However, identification of many thousand possible REST-target genes, mostly based on indirect criteria, and demonstration of their operative dependence on the repressor have been established for only a relatively small fraction. In the present study, starting from our recently published work, we have expanded the identification of REST-dependent genes, investigated in two clones of the PC12 line, a recognized neuronal cell model, spontaneously expressing different levels of REST: very low as in neurons and much higher as in most non-neural cells. The molecular, structural and functional differences of the two PC12 clones were shown to depend largely on their different REST level and the ensuing variable expression of some dependent genes. Comprehensive RNA-Seq analyses of the 13,700 genes expressed, validated by parallel RT-PCR and western analyses of mRNAs and encoded proteins, identified in the high-REST clone two groups of almost 900 repressed and up-regulated genes. Repression is often due to direct binding of REST to target genes; up-regulation to indirect mechanism(s) mostly mediated by REST repression of repressive transcription factors. Most, but not all, genes governing neurosecretion, excitability, and receptor channel signaling were repressed in the high REST clone. The genes governing expression of non-channel receptors (G protein-coupled and others), although variably affected, were often up-regulated together with the genes of intracellular kinases, small G proteins, cytoskeleton, cell adhesion, and extracellular matrix proteins. Expression of REST-dependent genes governing functions other than those mentioned so far were also identified. The results obtained by the parallel investigation of the two PC12 clones revealed the complexity of the REST molecular and functional role, deciphering new aspects of its participation in neuronal functions. The new findings could be relevant for further investigation and interpretation of physiological processes typical of neurons. Moreover, they could be employed as tools in the study of neuronal diseases recently shown to depend on REST for their development.

## Introduction

Since its discovery 20 years ago, the transcription repressor REST (RE-1 Silencing Transcription factor, otherwise called NRSF) has attracted continuous attention for its critical role in neuronal differentiation (Ballas and Mandel, 2005; Ooi and Wood, 2007; Johnson et al., 2008). Repression by REST occurs either by direct binding to specific DNA sequences included in various regulatory regions of target genes, or indirectly via the involvement of other transcription factors. The level of REST is high in stem cells and in early phases of neuronal precursor differentiation. At later stages the level of the repressor drops due to the increase of its proteasome turnover (Ballas and Mandel, 2005; Ooi and Wood, 2007; Johnson et al., 2008). This drop eliminates or attenuates the repression of REST target genes, contributing substantially to the specificity of the neuronal cell phenotype.

The number of REST target genes is still debated. The first ~2000 genes were proposed because of their positivity for RE-1, a DNA sequence of possible REST binding (Bruce et al., 2004; Wu and Xie, 2006; Johnson et al., 2007). More recently, thousands of additional genes, possibly REST-dependent but RE-1-negative, have been identified based on integrated computational analyses of available ChIP-Seq datasets, carried out mostly in non-neural cells (Otto et al., 2007; ENCODE Project Consortium et al., 2012; Johnson et al., 2012). So far, however, only a fraction of the above genes have been validated as REST-dependent. Which other RE-1-positive and negative genes operate, and the control of the repressor, remain unknown.

Identification of REST-dependent genes has been attempted also by differential expression analyses of two or more neuronal populations, distinct for state of development or functional activity. One of these analytic studies has been carried out in human pluripotent stem cells (iPSCs) differentiated *in vitro* (Rockowitz et al., 2014), i.e., in multiple populations that include neuronal subtypes at various stages of maturation together with neuronal progenitors. The results of this study have led to the identification of a moderate number of target genes encoding for various K^+^ channels (voltage-gated and not) and a few G protein-coupled receptors, governing structures, and general properties of neurons such as synapses (Rockowitz et al., 2014). Another study, dealing in this case with cortical neurons, has investigated the effects of few fold increases of REST induced by long-term treatment with kainate, a glutamatergic agonist. Out of the over 400 RE-1-positive genes investigated, only 39 encoding for voltage-gated and receptor channels, transcription factors, signaling proteins, and some protein kinases (PKs) were found to undergo significantly decreased expression (McClelland et al., 2014). Likewise, the expression of only few genes was found to decrease in the hippocampus upon REST increase induced by aging in healthy animals (Liu et al., 2014).

In conclusion, the REST dependence of many possible target genes, both positive and negative for RE-1, remains to be established. It should be emphasized, however, that the identification of REST-target genes can be attempted not only in populations of neurons, but also in neural cell lines largely employed, in the past and at present, as neuronal models. Among these lines the best known model is PC12 (Sombers et al., 2002; Martin and Grishanin, 2003; Ravni et al., 2008), a line of adult neural cells isolated from a rat pheochromocytoma (Greene and Tischler, 1976). The very low levels of REST typical of most PC12 clones, defined as wild type PC12 (wtPC12) clones (Bruce et al., 2006; D'Alessandro et al., 2008), are associated to numerous neuron-like processes including neurosecretion and NGF-induced neurite outgrowth (Greene and Tischler, 1976). However, in a few other PC12 clones, characterized by lack of neurosecretion and other neuronal properties, the REST levels, spontaneously much higher than in wtPC12, approach the levels typical of many non-neural cells (Pance et al., 2006; D'Alessandro et al., 2008). Interestingly, the defects of high REST PC12 (hrPC12) are attenuated upon the decrease of the level and/or the function of the repressor. Moreover, phenotypic properties of hrPC12 are induced in wtPC12 by the increase of their REST levels (D'Alessandro et al., 2008; Tomasoni et al., 2011). Based on these findings, most differences between wt and hrPC12 clones have been attributed to their different levels of REST (D'Alessandro et al., 2008). The comparative investigation of wtPC12 and hrPC12 clones appears therefore particularly advantageous, compared to other neural cell lines, for the identification of target genes governed by REST in neural cells.

The first, comparative analysis of PC12 clones, carried out by an old microarray technique prior to the demonstration of their different REST levels, had already identified in the hrPC12 cells two groups of ~170 genes significantly repressed and up-regulated compared to the wtPC12 cells (Grundschober et al., 2002). The studies have now been extended by the up-dated, highly sensitive RNA-Seq approach. In a first bio-informatic analysis of the obtained datasets, we found that, out of a total of ~13,700 genes (Table S1), almost 900 genes of hrPC12 cells exceeded significantly a cut-off of −2 log2 fold difference with respect to wtPC12 cells, while almost 900 genes exceeded significantly a cut-off of 2 log2 fold. Because of their greatly different expression in the two clones, the two groups of genes were identified as repressed and up-regulated primarily by REST. Previous investigation of these genes, carried out by the combination of RNA-Seq with ChIP-Seq enrichment analyses, had revealed REST repression and up-regulation to depend on different mechanisms, primarily direct in the first and almost always indirect in the second case (Garcia-Manteiga et al., 2015).

In the present work we have confirmed, strengthened, and greatly expanded our previously reported data (Garcia-Manteiga et al., 2015). Specifically, we have pursued the mechanistic investigation and carried out the first identification and functional analysis of the genes repressed and up-regulated in the hrPC12 versus the wtPC12 clones. The results, validated by RT-qPCR analyses and correlated to the expression in the clones of the encoded proteins, were employed to characterize the role of REST, working alone and in cooperation with other transcription factors (Ballas and Mandel, 2005; Ooi and Wood, 2007; Schneegans et al., 2009; Testa, 2011; Di Croce and Helin, 2013; Lund et al., 2015), in a population of target genes much larger than the population known until now. Taken together, our results have deciphered new aspects of the repressor action. Many of the proteins encoded by target genes appear involved in the development and function of critical properties of neurons. In addition, at least some of these genes could be involved also in the pathogenesis of various brain diseases recently shown to depend on REST for numerous, interesting aspects (Ooi and Wood, 2007; Schonrock et al., 2012; McClelland et al., 2014; Baldelli and Meldolesi, 2015). Most likely, therefore, the identification in PC12 cells of the genes dependent on REST for expression will be precious for further studies in various areas of cellular neurosciences.

## Materials and methods

### RNA sequencing and data analysis

The RNA, extracted with the RNeasy Mini Kit (Qiagen, Valencia, CA) from the two, carefully washed clones of PC12, wtPC12, and hrPC12 (previously referred to as PC12-27, D'Alessandro et al., 2008), was analyzed in duplicate with the Agilent 2100 Bioanalyzer (Agilent Technologies, Santa Clara, CA). Libraries, prepared starting from 2 μg of RNA/sample with the Illumina TruSeq RNA Sample Prep kit v2 procedure, were quantified by the Qubit BR assay (Life Technologies, Illkirch, France) and the Agilent 2100 Bioanalyzer, and sequenced on the Illumina HiSeq 2000 platform. On average we obtained about 90 million 100 bp PE (paired-end) reads per sample. Quality control of the obtained reads, and alignment to the rat reference genome (RGSC3.4/rn4) were performed using FASTQC suite with default parameters (FastQC, a quality control tool for high throughput sequence data, http://www.bioinformatics.babraham.ac.uk/projects/fastqc/) and the TopHat aligner (Trapnell et al., 2009). Gene expression read counts were exported and analyzed in R to identify differential expressed genes (DEGs), using the DESeq Bioconductor library (Anders and Huber, 2010). Genes with a baseMean value for all samples of < 5 or showing 0 reads as baseMean in either wtPC12 or hrP12 cells were filtered out to avoid infinite and 0 values of log 2-fold changes. *P*-values were adjusted using a threshold for false discovery rate (FDR) ≤ 0.01 (Benjamini and Hochberg, 1995). Genes listed as DEGs are reported in Table S1. Genes additionally filtered for absolute values of |log2 FC| > 2 (total 1770), were used for further analysis. Density and Volcano plot analyses, performed in R and heatmaps of expression values, were plotted with the pHeatmap library. Raw data are available through Gene Expression Omnibus (GEO) (http://www.ncbi.nlm.nih.gov/geo/query/acc.cgi?acc=GSE59946). Further details about the methods employed can be found in Garcia-Manteiga et al. (2015).

### Putative targets of transcription factor analysis

Metacore™ platform^1^ (http://thomsonreuters.com/site/systems-biology/, version 6.15, build 62452) was used (Table S2) for the analysis of transcription factor targets. Shortest path, algorithm-based networks were built using the ChIP-Seq data and interactions contained in the Metacore database. Components of REST/coREST/Sin3ab and PCR complexes were linked as input (*From*) to the genes modulated (repressed and up-regulated, |log2FC| > 2) in hrPC12 cells as output (*To*). The interactions in such a network were employed to define the genes that could be repressed by one or the other complex by using the union list of genes interacting with any of the components of each complex. The interactions in shortest path networks from transcription factors repressed in hrPC12 cells, focused to target genes not included in the network of REST/Polycomb, were used to construct networks (see Supplementary Information for details). For interaction networks, both repressed and up-regulated genes were introduced as an input to the “Direct Interactions Network” algorithm of Metacore, showing in the database the interactions that link all modulated genes directly (shortest path = 1). For further detailed information see Garcia-Manteiga et al. (2015).

### Gene function

#### Manual annotation

The annotated gene symbol from Rat Genome (rn4) dataset was used to search for known functions using PubMed and NCBI gene databases. Rat genes without an official symbol were identified by their human orthologous using the Ensembl database of gene annotations (www.ensembl.org). For genes whose function was predicted by homology to human or mouse genes, the annotation of the orthologous human gene symbol was given as an independent line or in brackets for cross referencing the Figures and Tables.

#### Gene-by-gene investigation

The repressed, up-regulated and unchanged data, reported in the Tables S3–S5, were investigated, gene-by-gene, starting from the information of the PubMed of NIH databases expanded by the investigation of the literature about the genes, the encoded proteins and their orthologous. The data thus obtained were then used to distribute the genes in 75 different groups, each corresponding to a distinct cell function.

### RT-qPCR

To validate the RNA-Seq data, expression of mRNAs was quantitatively assessed by RT-qPCR. Primer design (Table S6) was performed using the NCBI primer design tool (http://www.ncbi.nlm.nih.gov/tools/primer-blast/). Reverse transcription (RT) was obtained by iScript Advanced Synthesis kit for RT-qPCR (Biorad), starting from 1 μg total RNA for each sample not treated with DNase. The absence of interference by genomic DNA carryover in RNA samples was tested setting up a no-RT control reaction by substituting the reverse transcriptase volume with water. RT-qPCR was performed using Sso Advanced Universal SYBR Green Supermix (Biorad) in a Viia7 real-time system (Applied Biosystem). Each sample was assayed in duplicate. Results were normalized to β2 microglobulin (NM_012512) and peptidylprolyl-isomerase H (XM_001073803.4).

### Western blotting

Replicates of lysates (60 μg of proteins in RIPA-buffer) obtained from two different passages of hrPC12 and wtPC12 cells were separated on 10% SDS-PAGE under reducing conditions and after blotting onto nitrocellulose membrane immunodecorated with the following antisera: anti-REST (Millipore, 07-579), anti-Shank 2 (SYSY, 162202), anti-Shank 3 (SYSY, 162302), anti-Slc17A7 (SYSY, 135302), anti-tubulin (Sigma, DM1A), anti-annexin1 (Santa Cruz, Slc-12740), anti-GAPDH (SYSY, 247002), anti-Fev (Abnova, H00054738-A01), anti-Kcnk3 (Chemicon, AB5250), anti-Ascl1/Mash1 (RDI, 24B7.2D11), anti-SNAP25 (Sternberger Monoclonals, SMI81), anti-GRIN1 (LSBio, B7013), anti-Eps8 (BD, 610144), anti-Dlg4 (Abcam, P78352), and revealed by means of Clarity™ Western ECL Substrate (Biorad, Italy). Images, acquired by a Chemidoc MP (Biorad, Italy) with an Image Lab 5.0 software, were exported as tiff files and assembled with Photoshop CS4. Quantitation of band intesity on unsaturated exposures was performed with the Volume tool of the Image Lab 5.0 software. The adjusted values of the proteins of interest were normalized on those of GAPDH or tubulin bands of the corresponding lanes.

### Statistical analysis

**Figure 1:**
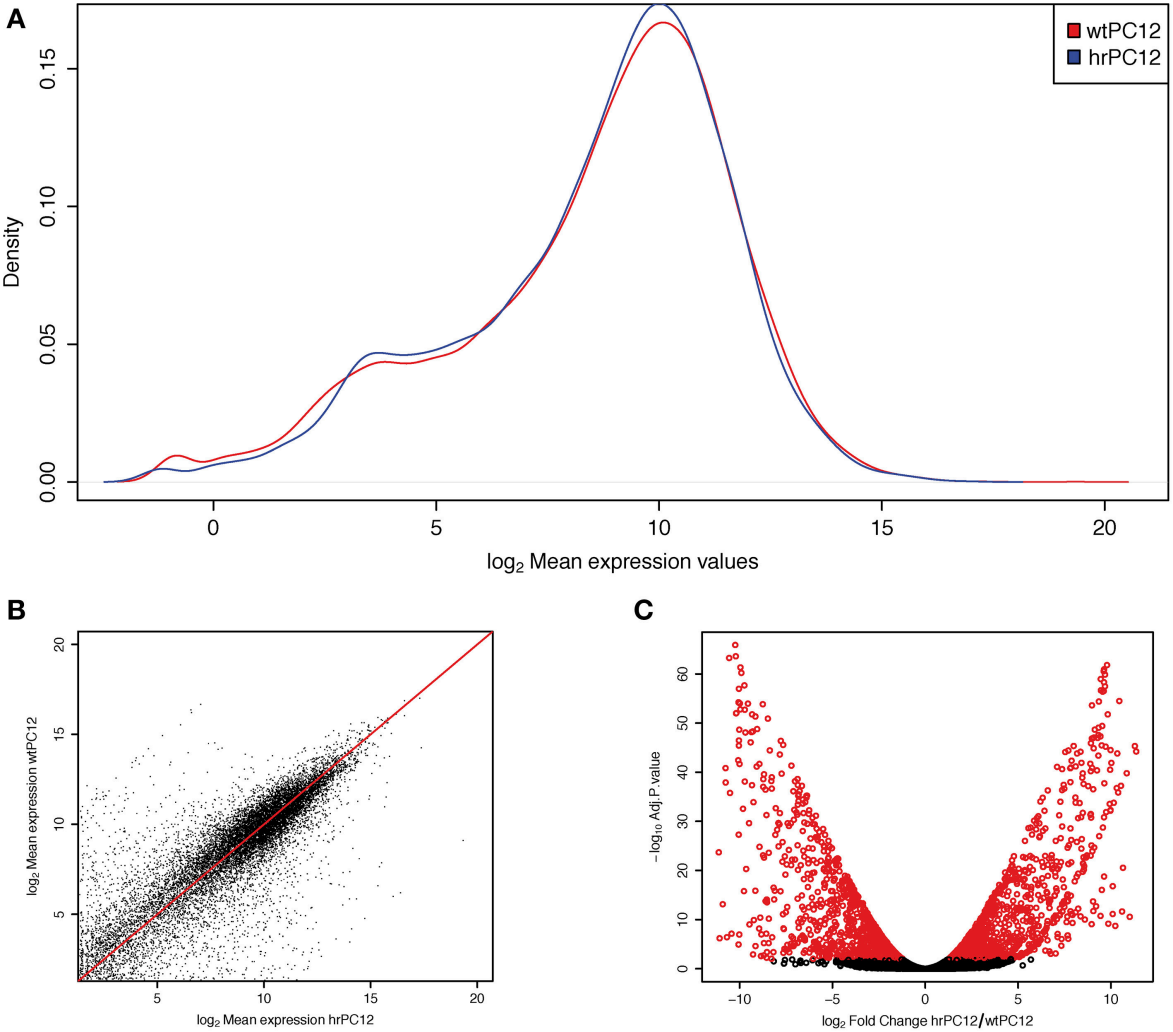
**Differential gene expression in wt and hrPC12 cells. (A)** Density plot of log2 mean abundance (reads mapped to a gene) in wtPC12 (red) and hrPC12 (blue) cells after normalization. **(B)** log/log mean abundance plot showing the distribution of genes in the two clones. Points distant to the red line reveal differential mean values. The distribution of the abundance and differences between the two clones are those expected for a RNA-Seq investigation. **(C)** Volcano plot of changes. Red circles represent significant gene differences, repressed (left) and up-regulated (right) in the hrPC12 cells (adjusted *p*-values < 0.01).

The statistical analysis of differential gene expression was made using the R/Bioconductor DESeq package. The quality metrics for RNA-Seq analysis shown in Figure 1 were made using DESeq and general R functions. For visualization of the expression levels in the heatmaps we used the vsd (variance stabilized transformation) tool in the DESeq package (**Figure 5B**) that, assuming a continuous normal distribution, allows downstream analyses (Garcia-Manteiga et al., 2015). Western blot results were analyzed statistically with GraphPad Prism v.5.

## Results

Part of the data concerning the RNA-Seq transcriptome investigation of the two PC12 clones, wt and hr, had been reported previously (Garcia-Manteiga et al., 2015). Out of the 13,700 genes analyzed, the comparative analysis in the two clones yielded two lists of repressed and up-regulated genes, each of almost 900 genes, exceeding the wtPC12/hrPC12 ratio values lower or higher than 2 log2 fold. Such down and up cut-off values, lower and higher than those commonly used in the literature, were chosen to obtain a conclusive identification of the REST-dependent genes. Critical discussion of the cut-off results will be included in further section of the Results. The genes with wtPC12/hrPC12 ratio values between the two cut-offs specified above were defined unchanged. The repressed, up-regulated and unchanged genes of hrPC12 are reported in Tables S1, S3–S5, and illustrated in Figures 1A–C.

### RNA-seq data validation by RT-qPCR

**Figure 2:**
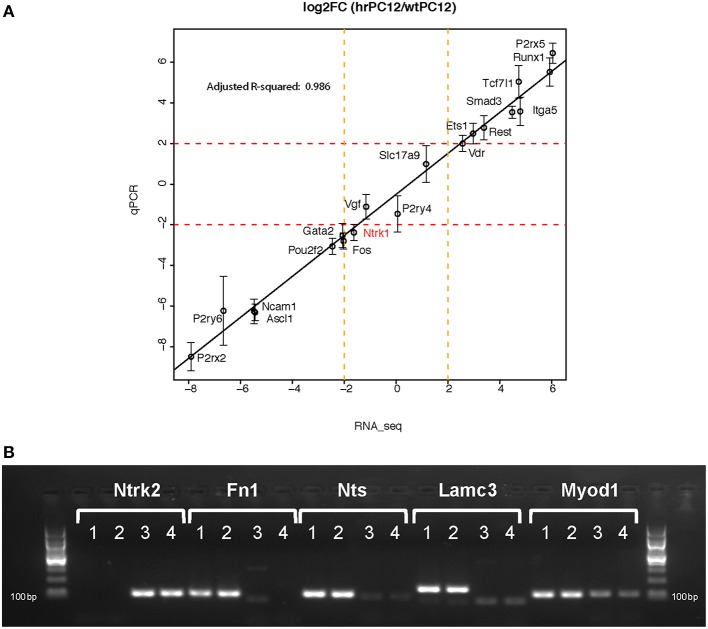
**RT-qPCR Validation**. The Figure compares the wtPC12/hrPC12 values of 24 genes, obtained by RT-qPCR duplicate samples, to the values obtained by RNA-Seq. Panel **(A)** illustrates the close matching results of 19 genes (qPCR values are mean of log2 fold change ± SD; Pearson correlation coefficient, *R*^2^ = 0.986). Panel **(B)** shows the PCR message results of 5 genes exhibiting in one clone values too little to be appropriately appreciated by RT-qPCR. The samples were only run on gels (lane 1 and 2 = hrPC12; 3 and 4 = wtPC12). The results confirm Ntrk2 (the TrkB receptor) to be repressed, and the other 4 genes to be up-regulated in the hrPC12 cells (RNA-Seq ratios: Ntrk2, −5.2; Fn1, 7.41; Nts, 9.44; Lamc3, 7.47; and Myod1, 8.18).

The validity of the differential gene expression values revealed by RNA-Seq in hrPC12 vs. wtPC12 clones was confirmed by consistent results. A validation approach was based on the comparison of the RNA-Seq with PCR data concerning the same genes. Values of 20 genes, repressed by RNA-Seq (asterisk-labeled in Table S1), were found to match closely the values previously obtained in the same cells by mRNA-qPCR (D'Alessandro et al., 2008; Klajn et al., 2009; Tomasoni et al., 2011; Mikulak et al., 2012). Additional specific validation was obtained by RT-qPCR of 24 genes made in duplicate, 8 of which repressed, 4 unchanged and 12 up-regulated (Figure 2). With 19 such genes, the wtPC12/hrPC12 ratio values amplified by RT-qPCR matched very closely those obtained by RNA-Seq (overall *R*^2^ correlation = 0.986; Figure 2A). The remaining 5 genes, including the Ntrk2 receptor of BDNF, were abundant in one clone and hardly detectable in the other by both RNA-Seq and RT-qPCR (Figure 2B).

### Correlation of the RNA-seq genes with the encoded proteins

The correlation of expressed genes with the levels of the encoded proteins was investigated by comparison of the RNA-Seq and western blot data. Among the 20 proteins investigated in previous studies (D'Alessandro et al., 2008; Klajn et al., 2009; Schulte et al., 2010; Prada et al., 2011; Tomasoni et al., 2011; Mikulak et al., 2012) a minor discrepancy was found only with Ntrk1, the NGF receptor TrkA. The RNA-Seq value, close to the negative cut-off (Figure 2), did not fit with the receptor protein values, similar in level in both wt and hrPC12 (Negrini et al., 2013). This result could be due to faster turnover of TrkA protein in wtPC12 than in hrPC12 cells.

**Figure 3:**
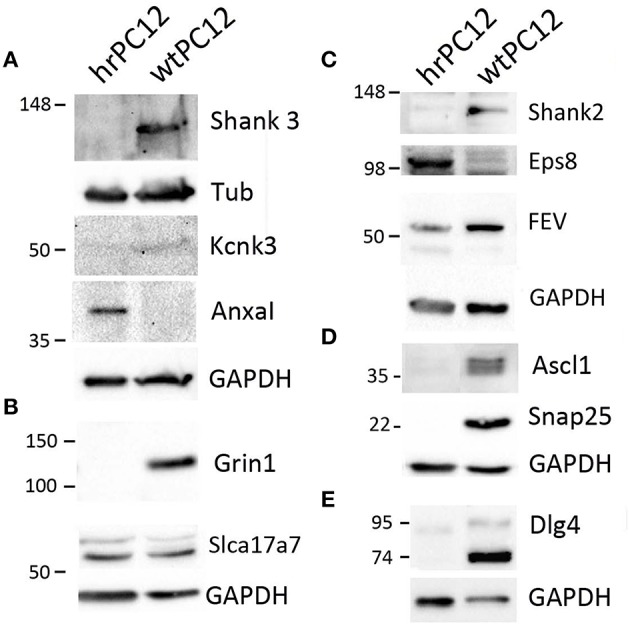
**Proteins encoded by genes differentially expressed in hrPC12 and wtPC12 cells**. Representative results of western blots of 11 proteins analyzed 2–4 times. Panel **(A)** shows the results of the repressed post-synaptic protein Shank3 and K^+^ channel Kcnk, with the up-regulated annexin1 (Anxal); controls with tubulin (Tub), and GAPDH. Panel **(B)** shows the results of the repressed Grin1, a subunit of the NMDA receptor, and the unchanged Slca17a7 Na^+^ transporter, with GAPDH control; Panels **(C–E)** show the repressed post-synaptic protein Shank2, transcription factor Ascl1, SNARE protein SNAP25, and MAGUK Dlg4, together with the moderately repressed FEV and the up-regulated synaptic protein Eps8. Controls with GAPDH.

**Figure 4:**
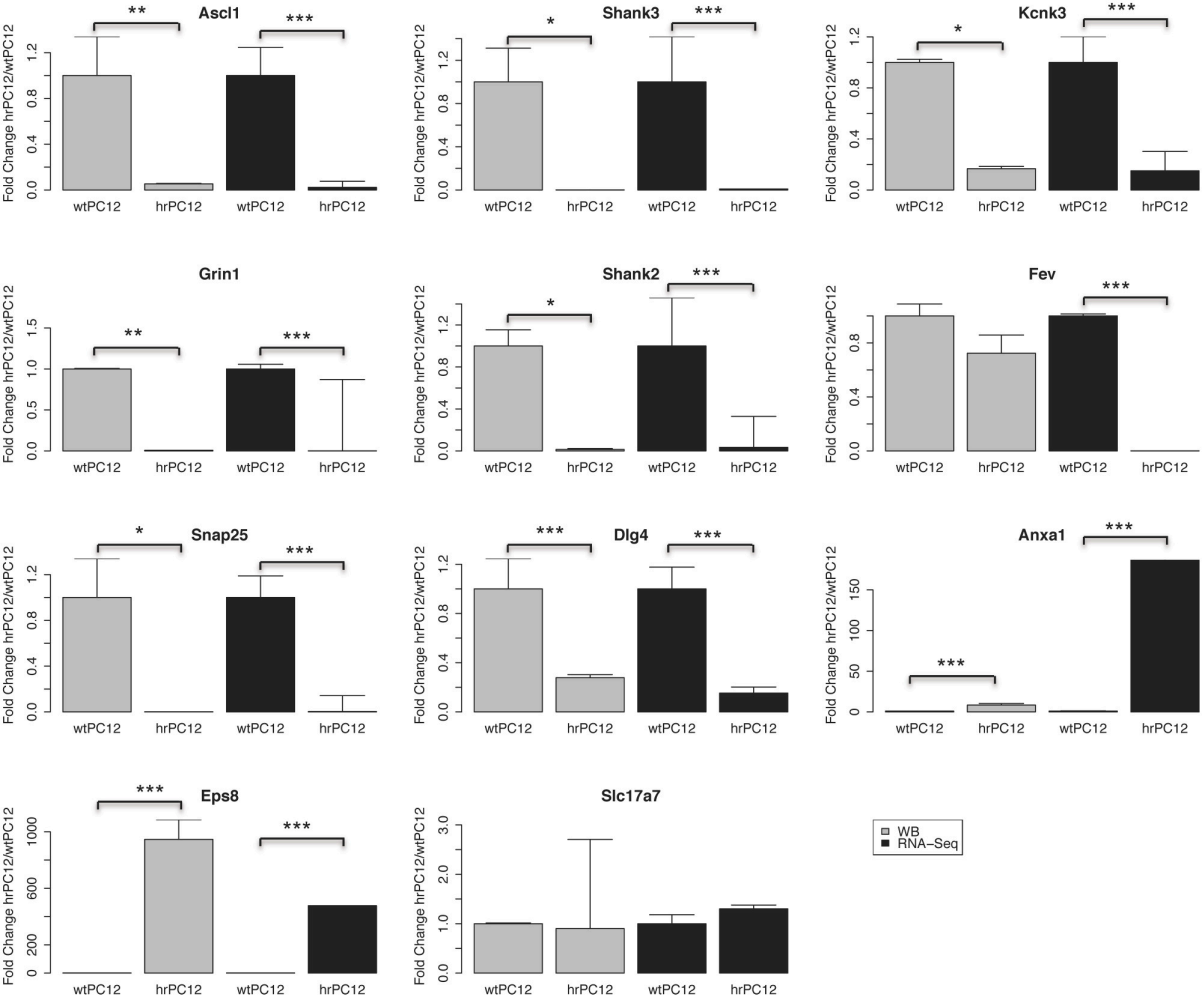
**Quantification of the western blot data of Figure 3**. The proteins encoded by 9 out of 11 genes: 7 down-regulated, 1 unchanged, and 1 up-regulated, exhibit levels parallel to the RNA-Seq values. Annexin 1 (Anxal) exhibits a significant up-regulation, however less marked than the up-regulated gene expression shown by RNA-Seq. The transcription factor protein Fev appears unchanged whereas its gene expression is strongly repressed. Mean fold change values are shown ± standard deviation. Western blot replicates employed ranged from 2 to 4. Statistical analysis was carried out by a paired *t*-test. For the RNA-Seq analysis, the adjusted *p*-value of the negative binomial (DESeq) was used. (^*^p < 0.05, ^**^p < 0.01, ^***^p < 0.001).

The correlation with the encoding genes was extended in the present study to the western blotting of 11 additional proteins, validated by the parallel markers Tubulin and GAPDH used as loading controls (Figures 3, 4). Analogously to their encoding genes repressed in hrPC12 (Figure 4), two proteins of the postsynaptic density, Shank 2 and 3; two proteins related to the NMDA receptor, the subunit Grin1, and the membrane-associated guanylate kinase (MAGUK) Dlg4; the transcription factor Ascl1, as well as the SNARE protein Snap25, already included in a previous investigation (D'Alessandro et al., 2008), exhibited very low levels. The same was found with the two-pore K^+^ channel protein, Kcnk3, in spite to its faint level (Figures 3, 4). The vesicle transporter Slc17a7 was unchanged as in gene expression, while the synaptic protein Eps8 was up-regulated in hrPC12 cells, similar to its encoding gene (Figures 3, 4). The up-regulation of another protein, annexin1 (Anxa1), was also significant, however lower than that of the encoding gene. Only the transcription factor Fev, down regulated in terms of gene expression, was in contrast unchanged as a protein (Figures 3, 4). These data suggest that, in the expression of 10 out of 11 proteins, transcription of the encoding genes predominates. In contrast, for the expression of the Fev protein, gene transcription is likely adjusted by translational and/or post-translational processes differentially operative in wt and hrPC12 cells.

### Repressed and up-regulated gene expression in the hrPC12 clone: Levels and mechanisms

In the two lists, a number of repressed and up-regulated hrPC12 genes were found to reach high or very high differential values with respect to wtPC12. The most repressed (>2000-fold) were A4galt encoding for the α1,4-galactosyltransferase, a type two protein glycosylating enzyme of the Golgi complex; Xkr7, encoding for a blood group precursor; and Wnk2, encoding for a cytosolic serine/threonine PK. The neural cell-specific genes most profoundly repressed (>1500-fold) were Gabrb3, encoding for the 3β subunit of the GABA-A receptor; Syt4, encoding for synaptotagmin 4, a protein involved in the exocytosis of dense-core vesicles (DCVs); and ChgB, encoding for the DCV neurosecretory protein chromogranin B (Table S3). In the same hrPC12 clone, 16 genes were up-regulated more than 1000-fold, and 171 from 100 to 1000 fold (Table S4). At the top of the list were three genes with hr/wt ratios >2000-fold: Aff3, encoding for a transcription factor preferentially expressed in the lymphoid tissue; Mpeg1, encoding for a perforin-like protein; and A2m, encoding for a protease inhibitor (Table S4). A fraction of the unchanged genes, with wtPC12/hrPC12 ratios between the two cut-offs values, are reported in Table S5.

As discussed previously by Garcia-Manteiga et al. (2015), REST action on gene expression can occur either by direct or indirect mechanisms. The first requires the binding of the repressor to specific DNA sequences, such as RE-1, included in regulatory regions or even in exons/introns of target genes; the second is based on the REST-dependent repression of other genes encoding for transcription factors that ultimately mediate the effects of REST. The present analyses have been carried out by creating a list of genes potentially governed by REST, including the RE-1 sequence, as well as other genes identified in a previously published study (Garcia-Manteiga et al., 2015) as genes present in the ENCODE ChIP-Seq (https://www.encodeproject.org) datasets from cell lines of neural origin. In addition, information about protein-DNA and protein-protein interactions was obtained from the Metacore™ (GeneGo)-curated database, dealing also with the REST repressor complexes (Sin3A/B and CoREST) and Polycomb repressor complexes (PRCs) (Tables S2, S7). In these analyses the possible REST-dependent genes included both RE-1-positive and RE-1-negative genes. Altogether, the genes of these analyses were several thousands.

**Figure 5:**
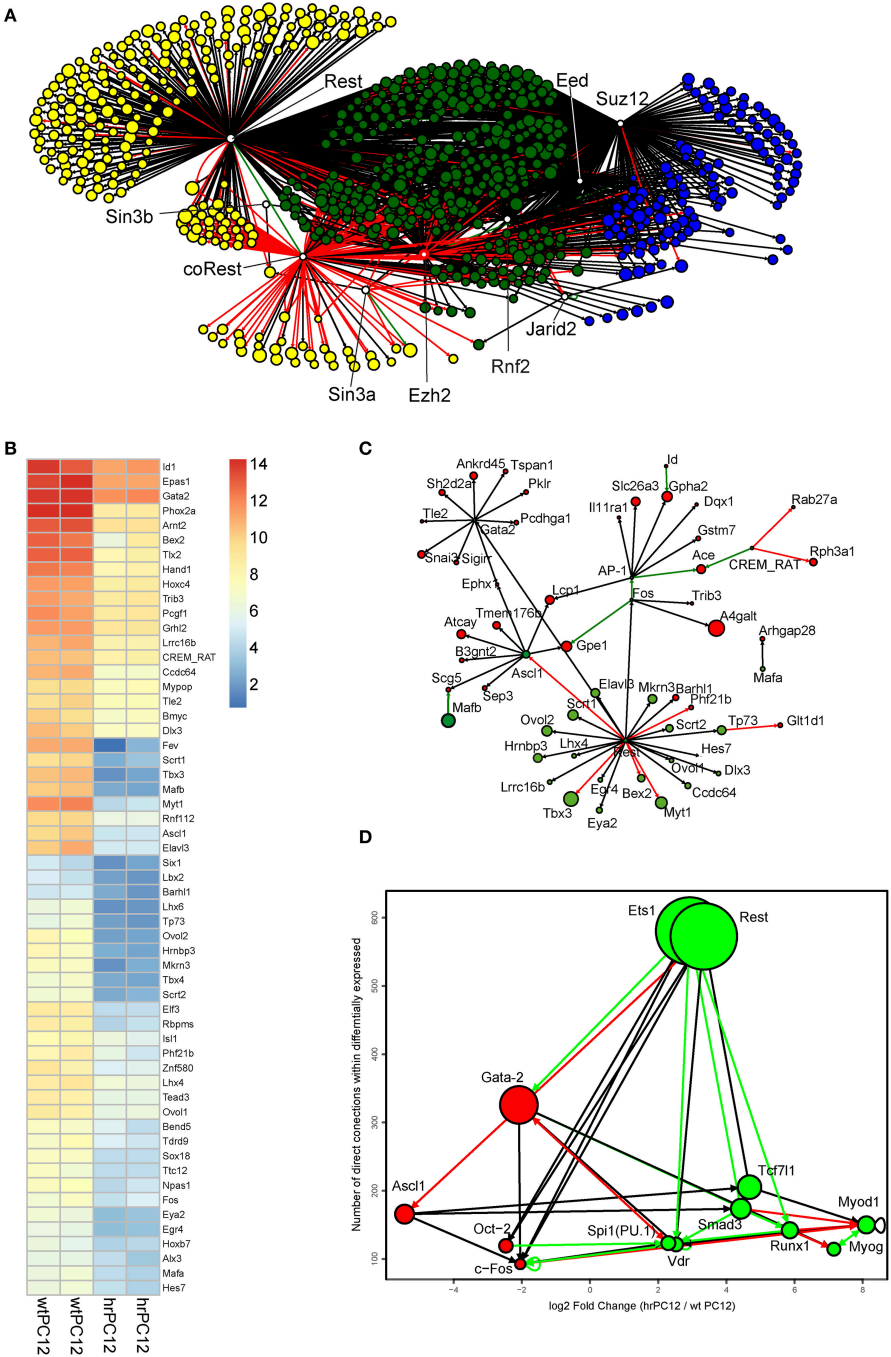
**Mechanistic insights into the REST gene expression control of PC12 cells. (A)** Shortest Path networks linking, through transcriptional regulation, the PRC 1, 2, and the REST complex proteins to genes repressed in hrPC12. The labeled circles represent the genes repressed in our dataset, shown in the Metacore™ databases and ENCODE dataset as putative targets of either the PRC 1-2 proteins (Suz12, EED, vEHZ2, and JARID2, blue circles) or the REST complex proteins (REST, Sin3B, Sin3A, and coREST, yellow circles). The putative targets of both REST and PRCs are shown in green. For repressed gene targets, the size of the node is proportional to the log2FC between hrPC12 and wtPC12 cells. The top 10-enriched Gene Ontology Biological Processes, carried out using the DAVID tool of enrichment, show genes under putative repression by REST only, PCR only or both. **(B)** Heatmap of the repressed transcription factor genes used in **(C)**. Statistical significance: cut-off at log2FC > ±2, adjusted *p*-values < 0.01. Levels of expression are shown after variance stabilization transformation and color code as in the scale shown. **(C)** Shortest path network linking the repressed hrPC12 transcription factor genes (green) of B to genes excluded from the network of REST/PRC-governed genes of **(A)** (red). In addition to Rest (included in the network to highlight the transcription factors that are among its potential direct targets), the most relevant genes of factors are Gata-2, Ascl1, and c-Fos (AP-1). The size of the nodes is as in **(A). (D)** Top connected hubs of a direct interaction network containing the repressed and up-regulated genes. The vertical position and size of the node indicates the number of connections with modulated genes. The horizontal position refers to their log2FC behavior. The red/left nodes correspond to the genes repressed in hrPC12 cells; the green/right nodes to the up-regulated ones. Black arrows indicate interactions predicted with unspecified effects; red and green arrows indicate interactions with experimental evidence for inhibitory and activatory effects, respectively.

Direct binding of REST is known to be important, but not always sufficient for gene repression. The process, in fact, is variable in different cell types and at various stages of development (Ballas and Mandel, 2005; Sun et al., 2005; Ooi and Wood, 2007; Johnson et al., 2008). An initial task of our work was to distinguish, among repressed hrPC12 genes, those affected directly from those affected indirectly. Out of the 886 genes repressed in hrPC12 cells, 571 (~64%) encoding not only for proteins but also for long–coding RNAs and miRNAs (Conaco et al., 2006; Wu and Xie, 2006; Johnson et al., 2009; Rossbach, 2011), appeared possibly governed directly by REST. In half of these genes (Figure 5A and Table S3), repression appeared possibly dependent on REST only, in the others REST repression is possibly operative in tandem with PRCs 1 and 2. For the remaining 315 (~36%) repressed genes the effect of REST appeared indirect (Table S3), possibly mediated by the control of PRCs or by the REST repression of stimulatory transcription factors (Figure 5B and Table S3). To identify the transcription factors involved in the process, we used interaction networks based on the information contained in Metacore protein-DNA and protein-protein interaction networks (Figure 5C and Table S2; see also Materials and Methods). Among the factors possibly involved in the indirect REST repression of numerous genes we found Ascl1 (known to promote neuronal differentiation), Gata2, c-Fos, and Oct2, each operative as a putative main hub, possible mediator in the indirect REST repression of numerous genes (Figures 5C,D; Otto et al., 2007; Castro et al., 2011).

REST potential target genes were investigated also in the population of genes up-regulated in hrPC12 (Table S4). Almost 40% of these genes were included in the lists of possible direct targets of REST, RE-1-positive, and negative. An interaction network (Figure 5D), however, confirmed almost all these genes to be up-regulated indirectly, most likely by the REST repression of other repressive factors. Among the genes possibly involved we found those encoding the transcription factor Smad3, potentially competent for indirect cooperation with REST. Additional possible hubs of up-regulation were Ets1, Tcf7l1, and Myod1 (Figure 5D).

The unchanged classification of the additional ~12,000 genes, including those previously reported by Johnson et al. (2012), was open to question being based on the high cut-offs employed. Nevertheless, their observation, reported in part in Table S5, was of interest. Among the unchanged genes away from the cut-offs we found a few encoding for components of complexes that mediate the repression by REST or PRCs (Tables S1, S5, and S6). These results exclude that the effects of these repressors are due to changes of these complexes. In contrast, some of the unchanged genes were found approaching, but not reaching, one of the two cut-offs. This appears the case of Vamp2, encoding for a vSNARE protein of neuronal cell exocytosis. Our previous RT-PCR data had already shown the VAMP2 gene to be repressed in hrPC12 cells, however only moderately (D'Alessandro et al., 2008). Additional unchanged genes approaching the negative cut-off were also related to functional areas rich in repressed genes, such as neurosecretion, channels, TKs, and adhesion proteins of the Ig-CAM family (Tables S1, S5). Conversely, some unchanged genes of hrPC12 were found to approach the positive cut-off. Future studies could establish the functional significance of the latter genes in neural and neuronal cell function.

### Functional relevance of differential gene expression

In order to extend the transcriptomic information to the functional level, we proceeded to a careful analysis of all repressed and up-regulated genes and of their intracellular distribution, based on the function of their encoded proteins. Tables S3, S4 illustrate the distribution of all repressed and up-regulated genes into 75 groups and sub-groups. Here we will focus on the properties of some such groups, highly relevant for the physiology of neurons. Examples of the genes of these groups are reported in Table 1.

**Table 1:** **Comparative transcriptome of hrPC12 and wtPC12 cells: Examples of genes encoding for proteins of various functional classes**.

	**log2FC (hrPC12/wtPC12)**	**Adj. *P*-Value**
**(A) NEUROSECRETION: PROTEINS TRIGGERING VESICLE EXOCYTOSIS**		
Cplx1	−6.33	4.8 × 10^−20^
Syt4	−10.56	5.5 × 10^−64^
Syt7	−7.20	2.7 × 10^−7^
Syt1^*^	−6.48	1.6 × 10^−6^
Stxbp3^**^	0.38	0.600
**(B) NEUROSECRETION: SECRETORY PROTEINS**		
Chgb^*^	−10.24	1.2 × 10^−66^
LOC100360310^***^	−9.96	4.6 × 10^−62^
Scg2^*^	−10.21	2.4 × 10^−64^
Pomc	1.49	0.086
Nts	9.44	4.9 × 10^−48^
**(C) EXCITATION AND SURFACE SIGNALING: VOLTAGE-GATED CHANNELS**		
Scn2a1	−3.04	5.1 × 10^−9^
SCN1A_RAT	1.16	0.1
Cacna1b	−8.44	6.1 × 10^−6^
Cacna1c	−4.33	2.6 × 10^−4^
Cacna1d	−4.50	4.3 × 10^−6^
Cacna1h	−5.12	0.002
Cacna1a	−0.06	1
**(D) EXCITATION AND SURFACE SIGNALING: G PROTEIN-COUPLED RECEPTORS**		
P2ry12	−10.00	2.1 × 10^−42^
Bdkrb2	−8.37	3.6 × 10^−45^
Chrm4	−1.74	2.5 × 10^−4^
Adra2b	−2.14	0.109
Gabbr1	−0.49	0.425
Htr2a	5.18	1.3 × 10^−12^
**(E) CELL ADHESION AND CELL MATRIX: PROTEINS OF THE IG-LIKE CAM FAMILY**		
L1-cam^*^	−4.10	1.1 × 10^−17^
N-cam1	−5.47	5.4 × 10^−5^
B-cam	−6.88	7.6 × 10^−26^
V-cam1	1.05	0.463
I-cam2	0.49	0.892
I-cam1	2.01	1.9 × 10^−5^
**(F) CELL ADHESION AND CELL MATRIX: METALLOPROTEINASES AND INHIBITORS**		
Mmp13	−9.96	1.6 × 10^−53^
MMP16_RAT	4.01	1.3 × 10^−13^
Thbs2	9.61	2.7 × 10^−61^
Timp3	7.00	2.5 × 10^−20^
Adam11	3.57	1.6 × 10^−9^

* *Similar values previously obtained in the same cells by qPCR*.

** *= Munc18-1*;

**** *= ChgA. (D'Alessandro et al., 2008; Klajn et al., 2009; Tomasoni et al., 2011; Mikulak et al., 2012). The genes shown are examples of the indicated functional classes. For additional genes look at the Tables S1, S3, S4*.

#### A. Gene expression

As already discussed, this is one of the areas in which REST control appears of the highest importance (Grundschober et al., 2002; Ooi and Wood, 2007). Here, for the first time, a comprehensive panel of transcription factors was reported. Forty-five genes were found repressed in hrPC12 cells, while 34 were up-regulated (Tables S3, S4). Interestingly, the genes coding for neural-specific factors were 21 among the repressed, and only four among the up-regulated. The genes coding for non-neural factors exhibited an almost opposite expression: 9 were repressed and 17, including the Rest gene itself, were up-regulated (Tables S3, S4). REST-dependent repressed and up-regulated genes coding for co-activators and modulators of transcription, for factors active in DNA binding and chromatin remodeling, RNA elongation and gene translation were also found (Tables S3, S4).

#### B. Neurosecretion

Neurosecretory clear vesicles and DCVs, abundant in wtPC12 cells, lack completely in hrPC12 cells. Such a defect is accompanied by, and most likely due, to the repression of genes encoding for specific proteins (Pance et al., 2006; D'Alessandro et al., 2008; Tables 1A,B; Table S3). Interestingly, Slc17a7, Slc17a9, and Sv2b, three genes of neurosecretory vesicles not repressed in hrPC12, (Tables S1, S4, and S5), encode for proteins operative also in other organelles of the cell (Miyauchi et al., 2006; Sawada et al., 2008; Yang et al., 2012).

Here we will report in sequence about the genes encoding for proteins that participate in the various steps of exocytosis (Jahn and Fasshauer, 2012; Kasai et al., 2012).

- **Priming**. Most genes encoding for proteins of this initial step of exocytosis: the genes of membrane proteins Cadps, Syngr3, Rims4, Sv2a, and Svop, and of the G proteins Rab3a and Rab3c, were all repressed in hrPC12 cells, some to very high extent (Table S3). Additional genes such as Munc13-1, Doc 2A, Rims2, and Syngr1, homolog to the genes coding for the priming proteins presented so far, were also reduced in hrPC12, however without reaching the negative cut-off (Table 1B, Table S3).
- **Triggering**. The proteins of this stage establish the conditions for the exocytic membrane fusion to take place. Expression of the Cplx1 gene, encoding for the SNARE complex binding protein complexin1, was profoundly repressed. The Stxbp3 gene, encoding for Munc18-1, was also reduced, however less than the −2 log2 cut-off (Table 1A; Table S3). Eight out of the 12 genes encoding for the Ca^2+^ sensory synaptotagmins, including Syts 4, 7, and 1, which play critical roles in the exocytosis of DCVs (Zhang et al., 2011), were greatly repressed (Table 1A; Table S3). The gene of Syt 9, another synaptotagmin known to operate in PC12 (Fukuda et al., 2002; Zhang et al., 2011), was however unchanged (Table S1).
- **Membrane Fusion**. Membrane fusion follows the direct, Ca^2+^-dependent establishment of the SNARE complex by three proteins, one of the vesicles, the other two of the plasma membrane (Jahn and Fasshauer, 2012; Rizo and Südhof, 2012). Of the two genes encoding vSNARE proteins, Vamp1 was repressed in hrPC12 cells, while Vamp2 approached the negative cut-off (Tables S3, S5). Additional 5 Vamp genes, unchanged in hrPC12, are known to encode for SNARE proteins operative in membrane fusions other than neurosecretion (Table S1). Likewise, repression of the plasma membrane SNARE genes was restricted to Snap25 and Stx1a and b, encoding for the proteins of neural exocytosis. The other 3 SNAP and 12 Stx genes were unchanged in hrPC12, while Stx11 was over-expressed (Tables S1, S3, and S4).
- **Secretory proteins**. These genes code for the proteins segregated within DCVs, released to the extracellular space by exocytosis (Jahn and Fasshauer, 2012). Of these genes, LOC100360310 (ChgA), ChgB, Scg2, 3, and 5, encoding for chromogranins and secretogranins; Dbh and Cpe, encoding for dopamine-β-hydroxylase and carboxypeptidase E; and the genes encoding for several peptide precursors, were all strongly repressed. In contrast, the genes encoding for other DCV secretory proteins, i.e., the granin VGF; Pomc and Pdyn, the genes of two opioid precursors, and NppA, the gene of the pro-natriuretic peptide A, were unchanged, while Nts, the gene of pro-neurotensin, was highly over-expressed in hrPC12 (Table 1B; Tables S3, S4 and S5). Additional secretory proteins are expressed by chromaffin and PC12 cells especially upon prolonged stimulation (Ait-Ali et al., 2010). In the hrPC12 clone, ~25% of their genes were repressed, ~25% over-expressed, and ~50% unchanged (Tables S1, S3, and S4).
- **Post-synaptic densities**. In the hrPC12 clone, many genes encoding the proteins of these densities, such as Shank 2 and 3, Homer 2, and PSD95, were repressed also at their protein levels (Figure 3 and Table S3).
- **Endocytosis**. In neural cells, exocytosis is matched by various forms of endocytosis (de Curtis and Meldolesi, 2012). The hrPC12 genes encoding for some of the endocytic proteins, including the Nsg1 gene of flotillin, Cav1 of caveolin 1, Ehd3 of the EHD3 ATPase, and Dnm1 and 3 of dynamins 1 and 3, were repressed (Table S3). In contrast Dnm2 and Dnm1L, encoding for dynamins 2 and 1L, and the genes encoding for various components of coated vesicles, were up-regulated (Table S4). Although likely modified in its various forms, therefore, the overall endocytosis appears largely preserved in hrPC12 with respect to wtPC12 (Cocucci et al., 2004).

#### C. Excitation and surface signaling

hrPC12 cells are known to be less excitable and different in surface signaling with respect to wtPC12. However, a comprehensive analysis of the role of REST in excitation gene expression had never been carried out.

- **Plasma membrane**. Many genes encoding for surface pumps of Na^+^, K^+^, H^+^ (Na^+^/K^+^; H^+^/K^+^), and Ca^2+^ (PMCA 2 and 3), were repressed in hrPC12 cells (Table S3). As far as channels, the negative control by REST (Ooi and Wood, 2007) was confirmed for the genes of Na^+^ voltage-operated channels (VOCs) (Table 1C). For Ca^2+^ channels, the repressed genes were those encoding for the α subunits of the L, N, and T VOCs. The only Ca^2+^ VOC with unchanged gene expression was the P/Q channel (Table 1C; Tables S1, S3). Several genes of K^+^ channels, VOC, and non-VOC, were also repressed (Table S3). Genes of the shaker and shab K^+^ VOC channels were however up-regulated, and this was the case of several non-VOC Cl^−^ channels, predominant in many non-excitable cells (Tables S1, S4).
- **Receptor channels, transporters**. Genes encoding for various subunits of nicotinic (Chrn), GABA-A and 5-hydroxytriptamine 3A receptors were all repressed in hrPC12. Other receptor channels, the glutamatergic NMDA, AMPA and kainate, and the purinergic P2X receptors were also affected, however less profoundly (Tables S3, S4). The genes of surface transporters are over 250. Of these only 13, encoding primarily for glucose and glutamate transporters, were up-regulated in hrPC12 (Table S4). Of the 31 repressed genes, most important were those encoding for the choline and catecholamine transporters (Table S3), necessary for the reuptake of these neurotransmitters following vesicle exocytosis.
- **G protein-coupled receptors** (**GPCRs)**. Only a few of the genes encoding for these receptors are repressed in hrPC12 cells, for example the purinergic P2ry12 and the bradykinin B2 receptors (Table 1D; Table S3). Up-regulated GPCR genes were more numerous, and many other genes were unchanged, including Chrm4, Adra2b, P2ry4, Grm2, and Grm4, encoding for muscarinic M4, the α2B adrenergic, the purinergic P2Y4, and the 2 and 4 forms of the GABA-B receptors, respectively (Table 1D; Tables S1, S4).
- **Tyrosine kinase (TK) and phosphatase receptors**. The genes encoding for many TK receptors: Egfr, Ntrk1 and Fgfr1 and 4, encoding for the EGF receptor; the NGF receptor TrkA; the FGF receptors 1 and 4, were unchanged in hrPC12 cells (Table S1). In contrast the genes encoding for other TK receptors, including Ntrk2 of TrkB, and the genes of three ephrins, were repressed. Additional genes, Fgfr3 (encoding for the FGF receptor 3), two ephrins and a few others, poorly known receptors, were up-regulated (Tables S3, S4). The genes of most protein tyrosine phosphatase receptors were unchanged in hrPC12, except for Ptprn (ICA512), involved in exocytosis, which was strongly repressed (Tables S1, S3). Unchanged were also many genes encoding for TK receptor ligands, however those of multiple EGF-like factors and PDGFB were repressed, while those of FGF7, VEGFC, and PDGFC, were up-regulated (Tables S1, S3, and S4).
- **Additional receptors**. Among the genes encoding for additional, structurally distinct receptors, strongly repressed in hrPC12 was Ngfr, encoding for the common neurotrophin receptor, p75^NTR^ (Table 2E). Other important repressed genes were Gfra2, Il22ra1 and Il11ra1, encoding for GDNFα2 and two interleukin receptors (Table S3). Up-regulated genes included Tlr3 and several genes of the TNF receptor family (Table S4). Finally, the genes of numerous receptor ligands, including various TNFs and growth factors, were up-regulated, whereas almost no such genes were repressed (Tables S3, S4).

#### D. Intracellular PKs; small G proteins

- **PKs**. The intracellular PK cascades, triggered by surface signaling, govern metabolic and functional activities of the cell. Repressed PK genes are of no major relevance in hrPC12, except for the Ca^2+^/calmodulin-dependent PKs IIβ and IV, involved in Ca^2+^ homeostasis (Table S3). A few up-regulated genes, encoding for Pak1, Pak3, and Rock2, regulated by G proteins, are highly important (Table S4).
- **G proteins**. In the hrPC12 cells, numerous genes of small G proteins and of their operational factors, GEFs and GAPs, were up-regulated. Additional up-regulated genes included a few oncogenes and regulators of the cytoskeleton (Table S4).

#### E. Cytoskeleton, cell adhesion and cell matrix

- **Cytoskeleton**. Cytoskeleton, a main target of intracellular signaling, interacts with the plasma membrane and the extracellular matrix (ECM). Numerous genes up-regulated in hrPC12 cells encode for cytoskeletal proteins specific of non-neural cell types, such as muscle fibers. Additional up-regulated genes were encoding for protein complexes known to reinforce actin fibers and their binding to the plasma membrane (Table S4).
- **Adhesion proteins**. Genes profoundly changed in hrPC12 were those encoding for adhesion proteins (Table 1E; Tables S3, S4). Repressed were the genes of the Ig-like protein superfamily, such as L1-cam, N-cam1, and B-cam, instrumental for neurite outgrowth, synapse formation and scouting of axonal pathways. The V-cam1, I-cam2, and Pe-camECM2 genes, encoding for three Ig-like superfamily proteins of general importance, were unchanged, while I-cam1 and a few others were up-regulated (Table 1E; Tables S3, S4, and S5). Genes encoding for cadherins and protocadherins were both repressed and up-regulated, while genes encoding for integrins, often involved in adhesion, and many other surface proteins were mostly up-regulated (Tables S3, S4).
- **ECM**. Considerable differences in gene expression emerged between the two PC12 clones also in relation to the extracellular matrix. In hrPC12 the up-regulated genes were 3-fold more numerous than the repressed genes and included Fn1, Lamc2, and 3, Thbs2 and 4 (encoding for fibronectin, laminins, and trombospondins) together with 14 forms of collagen (Tables 3C–E) and a number of metalloproteinases and their endogenous inhibitors of the ADAM, MMP, and TIMP families (Table 1F; Tables S3, S4).

## Discussion

Previous studies had already demonstrated the importance of REST in neuronal cells, not only in the course, but also upon completion of their differentiation. In particular, the typical low levels of REST were shown to increase in neurons in response to various long-term treatments, such as depolarization, kainate stimulation, and hypoxia (Calderone et al., 2003; Pozzi et al., 2013; McClelland et al., 2014; Baldelli and Meldolesi, 2015). Moreover, changes of neuronal REST levels had been reported during human aging, in various forms of cancer, and in several brain diseases, including Alzheimer's disease and epilepsy (Liu et al., 2014; McClelland et al., 2014; Baldelli and Meldolesi, 2015). In view of the relevance of REST in brain physiology and diseases it has become important to identify the genes governed by changes of the repressor, that in some cases have been reported to induce protection of neurons, in other cases to reinforce the toxicity of other treatments and diseases (Calderone et al., 2003; Pozzi et al., 2013; McClelland et al., 2014; Baldelli and Meldolesi, 2015). A fraction of the genes involved in these processes had been identified. However, attempts to expand the identification, carried out by various approaches such as the analysis of differentiated stem cells; of mature neurons long-term-treated with kainate, and of neurons aged in the brain, yielded information about relatively small numbers of genes (Liu et al., 2014; McClelland et al., 2014; Rockowitz et al., 2014).

Here we report about a comprehensive study carried out by RNA-Seq analysis of two clones of PC12 spontaneously expressing different levels of REST. The PC12 neural cell line is widely envisaged as a neuronal model (Sombers et al., 2002; Martin and Grishanin, 2003; Ravni et al., 2008). The validity of our results has been demonstrated by a close agreement with previous and present qRT-PCR results concerning 44 genes, part of which chosen at random. The functional relevance of the results, on the other hand, has been documented by the correlation between the expression of genes and the levels of 30 encoded proteins, known in part from previous studies and further expanded in the present investigation. Moreover, previous studies had demonstrated that changes of REST, induced in one clone to a level (or an activity) close to those of the other clone, were accompanied by parallel changes of the expression of a few target genes and of the general cell phenotype (D'Alessandro et al., 2008; Tomasoni et al., 2011). The structural and functional differences between the two clones appear therefore to depend largely on the difference of their REST concentration (Pance et al., 2006; D'Alessandro et al., 2008). Such difference approaches the differential values between neural and non-neural cells, i.e., it is ~10 fold larger than the changes induced in neurons by the long-term treatments mentioned above. From the functional point of view, the differential REST levels of the two clones most likely affect the expression not only of the high affinity target genes modified during the REST oscillations (Pozzi et al., 2013; McClelland et al., 2014; Baldelli and Meldolesi, 2015), but of a larger fraction of the REST-dependent genes.

The cut-offs of the hrPC12/wtPC12 ratios, employed to distinguish the genes repressed and up-regulated in hrPC12, were fixed at values (±2 log2 fold) already employed in the study of Garcia-Manteiga et al. (2015). These values, distinctly larger than those employed in previous studies by others, were chosen to focus especially on the genes governed most strictly by REST. Within the two groups of possible REST-dependent genes, the wtPC12/hrPC12 ratios varied from the cut-off values to thousand folds. Most genes known to be really REST-dependent were found to exhibit ratios larger than the cut-offs. Only few such genes did not exceed, but only approached the cut-offs.

In summary, because of their unique properties the two PC12 clones gave us the chance to investigate, in a valuable model, various aspects of the REST action. The specific results obtained are illustrated in detail in the Figures and Supplementary Tables of the paper. Here we will focus on two themes that studies by others had never taken into detailed consideration. The first theme deals with the mechanisms by which REST governs the repressed and up-regulated genes; the second, with specific properties of neuronal function revealed by gene repression and up-regulation.

### REST-dependent gene expression: Direct and indirect

In the literature, the REST-dependent genes have been often identified as genes repressed by the direct binding of the repressor. For REST-dependent up-regulation, however, the mechanism of the process remained largely undefined. Recently we had started to investigate the problem by the extensive, comparative analysis of the hrPC12 and wtPC12 clones. The approach employed was the combination of RNA-Seq with a ChIP-Seq Enrichment analysis, carried out using in parallel data from the ENCODE ChIP-Seq (ENCODE Project Consortium et al., 2012; Johnson et al., 2012) and from the Roadmap Epigenomics (Garcia-Manteiga et al., 2015; Roadmap Epigenomics Consortium et al., 2015). In the hrPC12 cells the number of repressed and up-regulated genes is almost equal, however the mechanistic results obtained were distinct. Direct binding of REST appeared in fact to account for the majority (~64%) of the repressions, while the remaining ~36% may be due to indirect processes; the up-regulated genes, that initially were suspected to include a fraction potentially competent for the direct binding, were in contrast mostly dependent on indirect mechanisms. However, knowledge of the complexity of these processes had remained at a preliminary stage (Garcia-Manteiga et al., 2015).

Gene transcription is often governed by the cooperation of multiple factors. This possibility is valid also for REST, which however is often assumed to operate independently from other factors. In the present study, evidence for putative cooperation has been shown only for PRCs. Other genes previously reported to cooperate with REST in other cells, i.e., the stimulatory Slug and TCF/Lef1 factors (Lund et al., 2015), were not found or were unchanged in PC12. It is likely, however, that other transcription factors and also additional agents, such as the non-coding RNAs and miRNAs, cooperate with REST in the govern of gene transcription.

A second form of cooperation with other transcription factors accounts for the indirect mechanisms of REST action. Indirect repressions were expected to depend on stimulatory transcription factors encoded by genes repressed directly by REST. The analysis of the latter genes identified four major candidates that may account for indirect repressions: Ascl1, Gata2, c-Fos, and Oct-2. On the other hand, when the indirect actions result in up-regulations, REST was expected to repress the genes of transcription factors that keep their target genes repressed. Also in this case the analysis identified four major candidates: Ets1, Smad3, Tcf7l1, and Myod1. It should be emphasized that the candidates of both groups could be identified because of the large number of their gene targets. Based on this property we have defined the above candidates as possible hubs in the gene interaction networks. The possibility that additional transcription factors operative in indirect repressions and up-regulations are also governed by REST cannot be excluded. However, additional factors have not been identified, possibly because of the low number of their repressed/up-regulated target genes.

### Functional relevance of REST-dependent genes

In view of its recognized master role in the differentiation of neurons and neural cells (Ballas and Mandel, 2005), it is not surprising that at least part of the REST-dependent genes operate in specific functional processes. The role of most genes differentially expressed in the two PC12 clones was identified and described in detail in the Results section. Based on functional criteria, these genes were all distributed in multiple groups and sub-groups of two specific Tables, S3 for the repressed and S4 for the up-regulated genes. In our opinion these, mostly descriptive results do not need a detailed discussion. Here we will concentrate on a few critical aspects of the most relevant REST-dependent functions revealed in the Results. The functions of additional genes and proteins, of possible interest in additional, specific fields of investigation, could be deduced from the comparison of the data reported in the Tables S1, S3, and S4 mentioned above.

In the transcription factor area, it is important to emphasize the differential properties of the REST-dependent genes expressed by the two PC12 clones. Even if the specificity of most transcription factors is not strict but varies in the various cell types, some factors are really neuronal. Many of the corresponding genes were found repressed, and only a few up-regulated in hrPC12. Such differences are expected to play key roles in the differential functional and phenotypic properties of the two PC12 clones.

Neurosecretion, a cell function typical of neurons and neural cells such as wtPC12, is in contrast lacking in hrPC12. The present results have extended the number and the characterization of the genes encoding for proteins specific of the various steps of the neurosecretory process. The genes of a few proteins known to be critical for the exocytosis of synaptic and DCV vesicles, such as VAMP2 and Munc18.1, although formally unchanged, could be considered repressed due to their adjacency to the negative cut-off. Unexpectedly, in contrast, the genes of four secretory proteins were really unchanged in the two clones, while the gene of another secretory protein was markedly up-regulated. Correlated with these findings was the observation that, of the genes of additional 70 secretory proteins that become abundant in wtPC12 upon prolonged stimulation (Ait-Ali et al., 2010), only 25% were repressed, 50% unchanged, and 25% up-regulated. The conclusion is that, in hrPC12 cells, the lack of classical neurosecretion does not imply the disappearance of secretory proteins. In order to be discharged, the secretory proteins maintained by hrPC12 cells were probably addressed to a pathway distinct from neurosecretion that remains to be identified.

A role of REST in cell excitability had already been documented (Ooi and Wood, 2007). Now we have shown that in hrPC12 cells the number of REST repressed genes encoding for VOCs of Na^+^, K^+^, and Ca^2+^ is much larger than previously reported by other laboratories. The genes of receptor channels (nicotinic, ATP, NMDA, and others), relevant in the post-synaptic membranes of cholinergic, glutamatergic, and other synapses, were also extensively or at least significantly repressed. In contrast, the genes of most GPCRs were unchanged or up-regulated. The state of other types of receptors was variable. For example, the genes of two TKs receptors, TrkB, and C and of the common neurotrophin receptor p75^NTR^, were all strongly repressed, whereas the gene of TrkA was not. Expression of several other growth factor receptors was unchanged or up-regulated. Taken together these results confirm the existence of profound differences in surface signaling related to the differential functions taking place in the two PC12 clones.

Our last issues focused on the differences in the cytoskeleton, mostly due to various proteins associated with actin filaments and microtubules, and on the general phenotype of hrPC12 cells, profoundly different from that of wtPC12 (Tomasoni et al., 2011). Among the genes of adhesion proteins, the hrPC12 repression was mostly limited to those encoding for the neuron-specific proteins of the Ig-like family, including L1-CAM and N-CAM. The genes of many other adhesion proteins were unchanged or up-regulated. A final type of genes, mostly up-regulated in hrPC12, were those encoding for ECM proteins, including metalloproteinases. These properties suggest the hrPC12 cells to be characterized by high surface dynamics.

## Conclusions

Previous studies had mostly emphasized the key roles played by REST in neurogenesis and differentiation of neurons (Ballas and Mandel, 2005; Ooi and Wood, 2007). During these studies the repressor was shown to govern gene expression via two mechanisms: direct repression, mediated by REST binding to specific DNA sequences in gene regulatory regions, and indirect mechanisms, possibly mediated by the repression of other transcription factors. In neurons, however, specific knowledge was restricted to only a fraction of direct REST target genes.

The present investigation, carried out by the combination of RNA-Seq with interaction networks, applied to the PC12 clones spontaneously expressing very different levels of REST, has given us the opportunity to expand the investigation. Repression of the genes governing previously identified processes, such as neurosecretion and excitability, is now accompanied by the lack of change and by the up-regulation of a few genes of the same processes; and by the predominant up-regulation of genes governing other processes: GPCR signaling, reorganization of the cytoskeleton, cell-to-cell interactions, and ECM dynamics. The present accumulation of many results has led us to hypothesize a number of criteria, including the following: that the concomitant REST-dependent expression of both repressed and up-regulated genes is an integrated mechanism by which the conversion of neural wtPC12 into less-neural hrPC12 cells is orchestrated; and that the present PC12 clone results could be useful for the development of further studies, including those in other types of cells such as neurons, where changes of REST are known to govern important functional processes. These and other criteria may be ultimately relevant for the investigation of several neurologic and psychiatric diseases, in which knowledge about REST-dependent genes could be ultimately relevant for the diagnosis and prevention, with stimulating perspectives for new forms of therapy (Schonrock et al., 2012; McClelland et al., 2014; Baldelli and Meldolesi, 2015).

## Financing of the work

Supported by the Telethon grant GGGP09066.

### Conflict of interest statement

The authors declare that the research was conducted in the absence of any commercial or financial relationships that could be construed as a potential conflict of interest.

## Footnotes

^1^Metacore from Thomson Reuters (NY) (2012) http://thomsonreuters.com/site/systems-biology/
