# Supplementary material for: REST-Governed Gene Expression Profiling in a Neuronal Cell Model Reveals Novel Direct and Indirect Processes of Repression and Up-Regulation
Source: Front Cell Neurosci. 2015 Nov 10;9:438. doi: 10.3389/fncel.2015.00438 (PMC4639699; doi:10.3389/fncel.2015.00438)
Supplement: Supplementary file 8 [file Presentation1.PDF]

Supplementary Information.

Identification of possible REST target genes.

The literature databases of genes bearing the RE-1 sequence in their regulatory areas exhibit only partial overlapping. Their combination leads to a total number of ~2,000 potential REST targets (Bruce et al., 2004). This number was further increased by the results of ChIP-Seq and other studies, documenting the physical binding of REST to DNA sequences (Table S2; Garcia Manteiga et al., 2015). Overall these studies demonstrated that the REST binding to its target genes changes considerably in the various cell types and in various physiological and pathological conditions of single cell types. Also with our hrPC12 clone, the overlap of the repressed genes with those reported in the literature as RE-1-positive (Table S2) was relatively low. Therefore, in order to identify the genes possibly dependent on REST, we decided to choose a set including all genes of rat, mouse or human, never indicated by empirical or bioinformatic means. In addition to the RE-1-positive genes we used as reference the list of gens identified by Johnson R. et al. (2012), that integrates the ChIP-Seq data of the ENCODE project, including genes from stem cells and three human cell lines of non-neural origin. These genes were identified, based on the ENCODE dataset server (http://genome.ucsc.edu/ENCODE/dataMatrix/encodeChipMatrixHuman.html), as the original *bed* files containing *broadPeaks* produced with MACS software (Zhang et al., 2008). Moreover, to expand the investigation to neural cells, we did add a list of possible REST target genes from cell lines of a new ENCODE dataset: ESC-H1 (from differentiated neurons, 2 samples), SK-N-SH (from a human neuroblastoma, 4 samples), PFSK1 (from an embryonic cerebral tumor, 4 samples) and U87 (from human primary glioblastoma, 4 samples). These datasets were processed by the *bedtools* suite (Quinlan and Hallk, 2010) to merge peaks showing a significant *p*-value of binding with REST in at least 2 samples coming from a total of 14 samples. The resulting peaks were intersected with human RefSeq annotations using a stringent criterion to reduce the peaks within the 5'UTR region of genes (hg19 RefSeq annotation table from UCSC, http://genome.ucsc.edu/cgi-bin/hgTables?command=start). The human genes of the list were then translated to their rat orthologs using Biomart (www.biomart.org), yielding a total of 4905 potential REST targets. This list was used as the ENCODE REST potential targets set in the Network analysis procedure. Finally, the data were complemented with other data coming from the network interactions database of Metacore, based on the publications about both the REST targets and the REST interacting proteins at the level of transcriptional regulation and protein-protein interactions (Table S2).

Network Analyses

Network analyses were made using the *Putative Pathway Modeling* of the Metacore network analysis suite, which allows specifying molecules serving as origins (*From*) and targets (*To*) of putative pathways. In our experiments REST and the other members of its complex, coREST, Sin3a and Sin3b, as

well as the proteins of the PRC 1 and 2 present in the Metacore database, were designated as *From*; the 884 genes repressed in hrPC12, as *To*. Since our investigation was restricted to connected nodes and shortest paths (n=1) in the network, the transcription complexes were expected to be linked only to their direct targets. In addition to the high-confidence, manually cured interactions, we also considered the "low trust" interactions of the Metacore algorithm. Such less-confidence interactions were also deduced from scientific papers. i.) They were based on manually cured data or experimental results obtained by techniques such as the yeast two-hybrid system, the ChIP-on-chip techniques, and others; and ii.) predicted from the analysis of nucleotide/protein sequences.

References

Bruce A.W., Donaldson I.J., Wood I.C., Yerbury S.A., Sadowski M.I,. Chapman M., Göttgens B., Buckley N.J. (2004) Genome-wide analysis of repressor element 1 silencing transcription factor/neuron-restrictive silencing factor (REST/NRSF) target genes. Proc. Natl. Acad. Sci. USA101: 10458-10463.

Garcia-Manteiga J.M., Bonfiglio S., Malosio M.L., Lazarevic D., Stupka E., Cittaro D., Meldolesi J. (2015) Epigenomics of neural cells: REST-induced down- and up-regulation of gene expression in a two clone PC12 cell model. Biomed. Res. Int. ID 202914. doi:10.1155/2015/202914.

Johnson R., Richter N., Bogu G.K., Bhinge A., Teng S.W., Choo S.H., Andrieux L.O., de Benedictis C., Jauch R., Stanton L.W. (2012) A genome-wide screen for genetic variants that modify the recruitment of REST to its target genes. PLoS Genet. 8: e1002624.

Quinlan A.R., Hall I.M. (2010) BEDTools: a flexible suite of utilities for comparing genomic features. Bioinformatics 26: 841-842

Zhang Y.1., Liu T., Meyer C.A., Eeckhoute J., Johnson D.S., Bernstein B.E., Nusbaum C., Myers R.M., Brown M., Li W., Liu X.S. (2008) Model-based analysis of ChIP-Seq (MACS). Genome Biol. 9: R137

Legends for Supplementary Figures and Tables.

Table S1. DESeq expression of all genes.

The genes in annotation analyzed (rn4_Ensembl_v69) were 25,810; the genes considered expressed (baseMean>5 & baseMean(hrPC12)>0 & baseMean(wtPC12)>0) were 13,713. The genes considered differentially expressed (|log2FC| > ±2 & adj.p-value < 0.01) were 1,770.

The wtPC12/hrPC12 ratio values of each gene are given in the F line. In the I line the unchanged genes are white, the 884 repressed genes (DOWN) are orange, the 886 up-regulated (UP) genes are green.

Table S2. Datasets and publications about the Metacore System.

The data of this Table were used in the identification of REST potential targets.

Table S3. Genes repressed in hrPC12 cells.

The almost 900 genes are distributed in 75 groups and subgroups based on the function of the encoded proteins, specified in the Table.

The genes colored yellow are possible targets of REST only, blue are possible targets of PRC, green of both and white of one or more unknown genes. The wtPC12/hrPC12 ratio values of each gene are given in the D line. A rapid definition of the function of all encoded proteins are given in the line F.

Table S4. Genes up-regulated in hrPC12 cells.

Distribution of the almost 900 genes is given as in Table S3.

As specified in the text, most (if not all) up-regulated genes are governed indirectly by REST. A possible direct control by the repressor (yellow), and a possible control by PRC can be suggested based on the data of Table S2. The white labeling excludes for some genes these possibilities. The wtPC12/hrPC12 ratio values and the protein functions are shown as in Table S3.

Table S5. Unchanged, RE-1 positive genes.

Out of the ~12,000 unchanged genes, this Table shows only a fraction of over 700, all RE-1-positive, chosen as in Johnson et al., 2012. The wtPC12/hrPC12 ratio values are given in the line C, and protein functions in the line E.

Table S6. List of qPCR primers used in Fig. 1.

These are the primers used for the validation experiments related to genes with the RefSeqIDs and PCR parameters.

Table S7. Differential expression of the genes encoding for proteins of the REST and PRCs complexes.

The Table illustrates the expression of genes encoding proteins involved in the operation of the complexes. The wtPC12/hrPC12 ratio values shown in the F line document that most of these genes are unchanged in hrPC12 cells.
